# Inflammasome activation controlled by the interplay between post-translational modifications: emerging drug target opportunities

**DOI:** 10.1186/s12964-020-00688-6

**Published:** 2021-02-24

**Authors:** Zhu Liang, Andreas Damianou, Elena Di Daniel, Benedikt M. Kessler

**Affiliations:** 1 Target Discovery Institute, Centre for Medicines Discovery, Nuffield Department of Medicine, University of Oxford, Oxford, OX3 7FZ UK; 2 ARUK Oxford Drug Discovery Institute, Nuffield Department of Medicine, University of Oxford, Oxford, OX3 7FZ UK; 3 Chinese Academy of Medical Sciences (CAMS), CAMS Oxford Institute (COI), Nuffield Department of Medicine, University of Oxford, Oxford, OX3 7FZ UK

**Keywords:** NLRP3 inflammasome, Post-translational modifications, Protein interaction, Signalling, Drug targets

## Abstract

Controlling the activation of the NLRP3 inflammasome by post-translational modifications (PTMs) of critical protein subunits has emerged as a key determinant in inflammatory processes as well as in pathophysiology. In this review, we put into context the kinases, ubiquitin processing and other PTM enzymes that modify NLRP3, ASC/PYCARD and caspase-1, leading to inflammasome regulation, activation and signal termination. Potential target therapeutic entry points for a number of inflammatory diseases focussed on PTM enzyme readers, writers and erasers, leading to the regulation of inflammasome function, are discussed.

**Video Abstract**

**Video Abstract**

## Background

Inflammasomes are multi-protein complexes that serve as important sensors to fend off pathogens and to resolve aberrant cellular physiology. Among the four types of inflammasomes, the NACHT, LRR and PYD domains-containing protein 3 (NLRP3) inflammasome subtype is the best characterised one. It can be activated by a variety of pathogens and sterile stimuli, which are termed pathogen-associated molecular patterns (PAMPs) or damage-associated molecular patterns (DAMPs). Inflammasome activation triggers caspase-1 dependent activation of pro-inflammatory cytokines, including IL-1β, as well as induction of an immunogenic type of cell death referred to as pyroptosis [1]. Compared with other inflammasome subtypes, aberrant NLRP3 activation is associated with a wide range of autoinflammatory and autoimmune diseases, such as gout, type 2 diabetes, atherosclerosis, Alzheimer’s disease and Parkinson’s disease [2]. Molecular insights into mechanisms of activation and how they are linked to disease pathogenesis remain limited and incomplete.

**Fig. 1:**
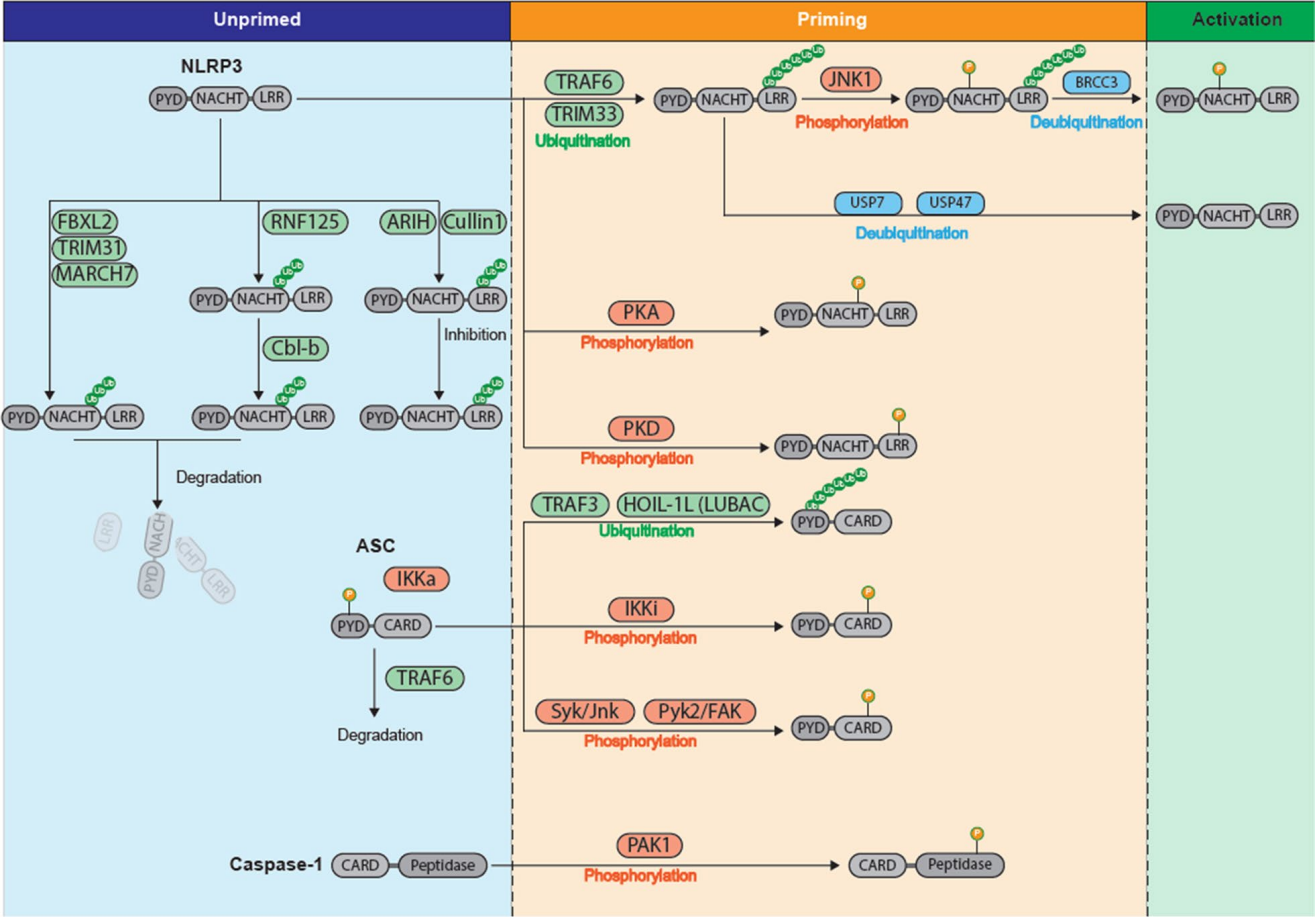
PTM regulation during NLRP3 inflammasome priming and activation

Canonical NLRP3 inflammasome activation is reported to require two signals (priming and activation). This “two-step stimulation model’’ was first proposed in 2009 by two groups [3, 4], suggesting that NLRP3 has to be primed before the initiation of complex assembly through the TLR (Toll-like receptor)-NF-κB signalling pathway (Fig. 1). The mechanism of action underlying priming has long been thought to be the transcriptional upregulation of NLRP3 and pro-IL-1β expression [3, 4]. This idea was challenged and refined by recent studies. For instance, Juliana et al*.* reported that 10 min treatment with lipopolysaccharides (LPS) was sufficient to prime the NLRP3 inflammasome in mouse bone marrow derived macrophages (BMDMs) without an increase in NLRP3 protein expression, suggesting that the effect of LPS does rely on post-translational processes other than transcriptional upregulation of NLRP3 [5]. Consistent with these data, it has been shown that NF-kB inhibitors, which are supposed to preclude NLRP3 transcription, had little effect on NLRP3 inflammasome activation in THP-1 macrophages [6]. Recently, it has been demonstrated that LPS priming increases NLRP3 protein by prolonging its half-life rather than via transcriptional upregulation [7]. Mounting evidence seems to suggest that non-transcriptional regulation is important in NLRP3 inflammasome priming, allowing rapid and tightly controlled innate immune responses to diverse stimuli. In addition to priming, the second signal, such as potassium efflux induced by the K^+^/H^+^ ionophore nigericin or ATP, has also been reported to regulate full activation of the NLRP3 inflammasome at non-transcriptional level, but the exact mechanism remains elusive [8, 9].

A number of post-translational modifications (PTMs) have been described to regulate the inflammasome [10]. Amongst the most extensively studied are ubiquitination and phosphorylation, which have been reviewed in detail elsewhere [11, 12]. These PTMs are thought to represent key switches in the priming process and utilized to get NLRP3 licensed for complex assembly. Other modifications, such as acetylation [13], ADP-ribosylation [14], nitrosylation [15] and ubiquitin-like conjugation (Sumoylation [16] and Neddylation [17]), have recently been reported to contribute to the regulation of NLRP3 inflammasome activation, but their significance still remains unclear.

In this review, we focus on the interplay between PTMs regulating the two stages of NLRP3 inflammasome activation. We discuss potential pharmacological molecules that target PTM enzyme readers, writers and erasers and their possible clinical applications in inflammatory diseases regulated by the inflammasome.

## PTM regulation of NLRP3 inflammasome components

Since it was initially discovered as a molecular scaffold [18], NLRP3 inflammasome is reported to include three main components: the sensor (NLRP3), adaptor (ASC/PYCARD) and effector (caspase-1). The assembly of the NLRP3 inflammasome relies on protein–protein interactions between the three main components, which is tightly regulated by post-translational modifiers. Although we would anticipate that each of the inflammasome components should be post-translationally modified at some stage, NLRP3, ASC/PYCARD and caspase-1 have been studied in detail (Fig. 1).

### NLRP3: combinatorial control by multiple PTMs

#### The ubiquitin system controls NLRP3 stability and priming

**Fig. 2:**
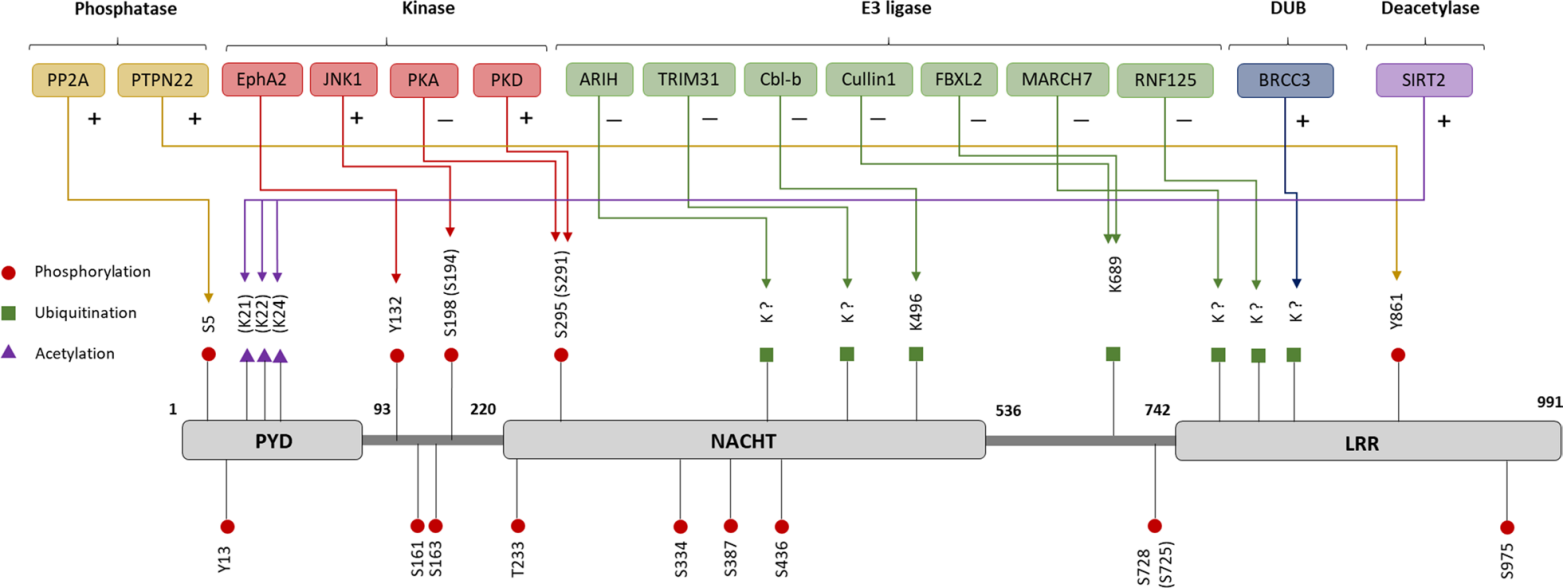
NLRP3 PTMs and the enzymes responsible for their regulation. Empirically validated enzymes which are responsible for the PTM regulation of NLRP3. “+” indicates that the enzyme promotes the activation of NLRP3 inflammasome, while “−” indicates repression of its function. Amino acid residues shown above refer to human NLRP3, those in brackets refer to mouse NLRP3 residues

There are 68 lysine residues in the human NLRP3 protein. However, only two (K689 [7] and K496 [19]) have been verified as ubiquitination acceptor sites via mass spectrometry or functional experiments (Fig. 2) [7, 19]. While there is limited empirical evidence of ubiquitination sites on NLRP3, possibly due to low basal NLRP3 expression under physiological conditions, the combination of molecular and pharmacological approaches has revealed the essential role of the ubiquitin system in controlling NLRP3 stability and priming.

NLRP3 ubiquitination was first identified in 2012, the first PTM identified on NLRP3 [5]. In the study, either the priming signal (LPS) or the activating signal (ATP) induced NLRP3 deubiquitination, and the non-selective deubiquitinase (DUB) inhibitors PR-619 and WP1130 completely prevented NLRP3 deubiquitination mediated by LPS or ATP [5]. It was concluded that the ubiquitination of NLRP3 might be controlled by different E3 ligases/DUBs acting at the priming and/or activation stages.

In the resting state, NLRP3 is usually highly ubiquitinated by both K-48 and K-63-linked poly-ubiquitin chains, and it is maintained at low expression levels mainly through proteasome-dependent degradation [5]. It has been reported that NLRP3 is ubiquitinated by seven E3 ligases, namely MARCH7, ariadne homolog 2 (ARIH2), tripartite motif-containing protein 31 (TRIM31), casitas B-lineage lymphoma proto-oncogene b (CBLB), F-box/LRR-repeat protein 2 (FBXL2), Cullin1 (CUL1) and RING finger protein 125 (RNF125) (Fig. 2, Table 1). All these seven E3 ligases act as negative mediators in the regulation of NLRP3 inflammasome, and most of them play an essential role in the TLR ligands-induced priming process. FBXL2, a component of SCF (SKP1-cullin-F-box protein) E3 ubiquitin-protein ligase complex, promotes poly-ubiquitination of NLRP3 at K689 site (in human, K687 in mouse NLRP3) via binding to W73 site, leading to its degradation [7]. TRIM31 directly binds to the PYD domain of NLRP3 and induces its K-48-linked poly-ubiquitination via the N-terminal RING domain [20], although another study did not report this (19). CBLB, a RING finger E3 ligase, has been shown to bind to the LRR domain and induce K48-linked poly-ubiquitination of the NACHT domain at K496 site (in human, K492 in mouse NLRP3), leading to NLRP3 degradation. The recruitment of CBLB relies on the K63-linked polyubiquitination of NLRP3 LRR domain by another RING finger E3 ubiquitin ligase-RNF125 [19]. Another study found that dopamine D1 receptor activation promoted K-48-linked ubiquitination and degradation of NLRP3 through the E3 ligase MARCH7, which was identified in the pull-down precipitate of NLRP3-flag by mass spectrometry [21]. In addition to degradation, poly-ubiquitination also affects functional activity of NLRP3 protein. ARIH2, which belongs to RBR (RING between RING fingers) family, was found to interact with NLRP3 and induce both K-48 and K-63-linked poly-ubiquitination, resulting in the repression of NLRP3 but not its degradation [22]. It is interesting to note that the depletion of ARIH2 was able to completely remove the ubiquitination of NLRP3 in THP-1 cells, which suggests that ARIH2 is an essential mediator of NLRP3 ubiquitination [22]. Another recent study reported that CUL1 inhibits the assembly of the NLRP3 complex by promoting K63-linked ubiquitination at K689 site, possibly disrupting complex formation by competing with the adaptor protein ASC [23].

**Table 1:** E3 ligases involved in ubiquitination of NLRP3 inflammasome components

E3 ligase	Activator	Substrate	Ubiquitylation site^a^	Molecular or cellular outcome	Linkage specificity	Refs.
FBXL2	LPS	NLRP3	K689	NLRP3 degradation	unknown	[7]
TRIM31	LPS/IL-1β	NLRP3	NACHT domain	NLRP3 degradation	K48-linked	[20]
MARCH7	Dopamine	NLRP3	LRR domain	NLRP3 degradation	K48-linked	[21]
CUL1	LPS	NLRP3	K689	NLRP3 ubiquitination/negative regulation	K63-linked	[23]
CBLB	LPS/ATP	NLRP3	K496	NLRP3 degradation	K48-linked	[19]
RNF125	LPS	NLRP3	LRR domain	NLRP3 degradation	K63-linked	[19]
ARIH2	LPS/ATP/Nigericin	NLRP3	NACHT domain	NLRP3 ubiquitination/negative regulation	K48/K63-linked	[22]
Pellino2	LPS	Unknown	Unknown	NLRP3 ubiquitination/positive regulation	K63-linked	[24]
TRAF6	Pam3csk4	Unknown	Unknown	NLRP3 oligomerization/positive regulation	unknown	[25]
HOIL-1L (LUBAC)	LPS	ASC	PYD domain	ASC linear ubiquitination/positive regulation	M1-linked	[34]
TRAF3	VSV^b^	ASC	K174	ASC ubiquitination/positive regulation	K63-linked	[33]
cIAP1/cIAP2/TRAF2	LPS	CASP1	unknown	CASP1 ubiquitination/positive regulation	K63-linked	[46]

^a^Amino acid residues shown above refer to human NLRP3, those in brackets refer to mouse NLRP3 residues

^b^VSV, vesicular stomatitis virus

In addition to negative regulators, two E3 ligases (Pellino2 and TRAF6) function to positively regulate NLRP3 activation (Table 1). A recent study reported that Pellino2 facilitates NLRP3 activation through K-63-linked poly-ubiquitination during the priming phase, although the mechanism remains unclear [24]. Another report found that TRAF6 positively regulates the oligomerization of NLRP3 via its E3 ligase activity, but the mechanism of action is largely unknown [25]. It should be noted that there is no evidence showing that NLRP3 is the direct target of either Pellino2 or TRAF6.

Up to date, three DUBs, including Lys-63-specific deubiquitinase BRCC36 (BRCC3), ubiquitin carboxyl-terminal hydrolase 7 (USP7) and ubiquitin carboxyl-terminal hydrolase 47 (USP47), have been implicated in the regulation of NLRP3 ubiquitination (Fig. 2, Table 2). Particularly, Benedicte et al*.* found that, knockdown of BRCC3 (a component of the BRISC complex) completely inhibited caspase-1 cleavage and IL-1β secretion. Mechanistically, BRCC3 promotes downstream inflammasome activation through specifically interacting with and deubiquitinating LRR domain of NLRP3 [9]. Another study found that pharmacological inhibition of USP7/USP47 prevented the formation of ASC specks and dramatically decreased both IL-18 and IL-1β release. Furthermore, the activity of USP7/USP47 was shown to increase in response to LPS and nigericin stimuli, which suggests a post-translational regulation of DUBs upon activation [8].

**Table 2:** DUBs involved in deubiquitination of NLRP3 inflammasome components

DUBs	Activator	Substrate	Ubiquitylation site	Molecular or cellular outcome	Linkage specificity	Refs.
BRCC3	LPS	NLRP3	LRR	NLRP3 deubiquitination	unknown	[9]
USP7	LPS	NLRP3	Unknown	NLRP3 deubiquitination	unknown	[8]
USP47	LPS	NLRP3	Unknown	NLRP3 deubiquitination	unknown	[8]
USP50	LPS/Nigericin	ASC	Unknown	ASC deubiquitination/positive regulation	K63-linked	[38]

#### Phosphorylation controls NLRP3 self-association and interacting networks

As one of the most frequent PTMs, phosphorylation dynamically controls multiple aspects of NLRP3 inflammasome components, including protein binding affinity, localization, and functional activity, at different stages. The signalling pathways involved in phosphorylation regulation have been reviewed elsewhere [11]. Thus, here we mainly describe more recent discoveries, and how phosphorylation interplays with other PTMs regulating inflammasome function.

Phosphorylation and dephosphorylation of NLRP3 have been shown to be an essential step for both priming and activation of the NLRP3 inflammasome. Hitherto, several phosphorylation sites, including S5, Y13, (Y132), S161, S163, S198 (S194), T233, S295 (S291), S334, S387, S436, S728 (S725), Y861, S975 (in human NLRP3, those in brackets refer to sites in mouse NLRP3), have been identified through affinity purification-mass spectrometry (AP-MS) or site-mutation functional experiments (Fig. 2 and Table 3).

**Table 3:** Kinases involved in phosphorylation of NLRP3 inflammasome components

Kinase	Activator	Substrate	Phosphorylation site^a^	Domain	Molecular or cellular outcome	Refs.
JNK1	LPS	NLRP3	S198 (S194)	Between PYD and NACHT	NLRP3 self-association and activation	[29]
PKD	Golgi dysregulation	NLRP3	S295 (S291)	NACHT	NLRP3 activation	[31]
PKA	Bile acids/PGE2	NLRP3	S295 (S291)	NACHT	Block NLRP3 activation	[27, 30]
EphA2	–	NLRP3	(Y132)	Between PYD and NACHT	Block ASC oligomerization	[32]
IKKα	LPS	ASC	(S16), (S193)	PYD and CARD	Translocation of ASC/negative regulation	[43]
IKKi	LPS	ASC	S58	PYD	Translocation of ASC/positive regulation	[43]
Syk/JNK	LPS	ASC	Y 146 (Y144), (Y187)	CARD	ASC oligomerization	[40, 44]
Pyk2/FAK	MSU^b^	ASC	Y146 (Y144)	CARD	ASC oligomerization	[45]
PAK1	LPS	CASP1	S376	Peptidase C14	Caspase-1 auto-cleavage	[49]

^a^Amino acid residues shown above refer to human NLRP3, those in brackets refer to mouse NLRP3 residues

^b^MSU, monosodium urate

Phosphorylation at sites S5 and Y861 (in human NLRP3) suppresses NLRP3 inflammasome activation (Table 4) [26–28]. In unprimed cells, S5 on NLRP3 is hyper-phosphorylated. Phosphorylated S5 disrupts the PYD-PYD interaction and inhibits the subsequent ASC recruitment to NLRP3. This suggests that dephosphorylation of S5 may be required for NLRP3 activation. Interestingly, the phosphatase PP2A has been shown to dephosphorylate S5, but it still remains unclear whether NLRP3 is the direct substrate of PP2A [28]. Similarly, Y861 phosphorylation was reported to inhibit NLRP3 activation. The tyrosine-protein phosphatase non-receptor type 22 (PTPN22) was shown to directly interact with NLRP3 and dephosphorylate Y861, leading to NLRP3 activation [26]. It is interesting to note that the Y861C mutation in humans is associated with chronic infantile neurologic cutaneous and articular syndrome (CINCA) [26].

**Table 4:** Phosphatases involved in dephosphorylation of NLRP3 inflammasome components

Phosphatase	Activator	Substrate	Dephosphorylation site^a^	Domain	Molecular or cellular outcome	Refs.
PP2A	–	NLRP3	S5	PYD	NLRP3 activation	[28]
PTPN22	–	NLRP3	Y861	LRR	NLRP3 activation	[26]

^a^Amino acid residues shown above refer to human NLRP3

Conversely, Jun N-terminal kinase-1 (JNK1) mediated S194 (in mouse NLRP3; S198 in human NLRP3) phosphorylation positively regulate NLRP3 activation. It was reported to be an essential priming step and required for NLRP3 self-association. Mechanistically, S194 phosphorylation might provide a receptor site for BRCC3 binding, which induces deubiquitination and promotes subsequent activation by the second stimulus. Notably, even though S194 phosphorylation is essential for priming, a NLRP3-S194D phosphomimetic mutant does not seem sufficient as a second signal to induce complex assembly [29].

In addition, phosphorylation of S295 (in human NLRP3; S291 in mouse NLRP3) was shown to be regulated by two kinases PKA and PKD. During the cAMP-induced NLRP3 inhibition, activated PKA directly phosphorylates NLRP3 at S295 and subsequently suppresses ASC oligomer formation [30]. Another study reported a completely opposing effect of S295 phosphorylation. Upon stimulation, PKD phosphorylates NLRP3 S295, which promotes downstream self-association of NLRP3 and releases it from mitochondria-associated membranes (MAMs) [31].

Recently, it was shown that phosphorylation of Y132 (in mouse NLRP3) in airway epithelial cells (AECs) interfered with ASC oligomerization and precluded IL-1β production. Mechanistically, Y132 phosphorylation is induced by Ephrin type-A receptor 2 (EphA2), which is selectively expressed in AECs and contributes to inhibiting the NLRP3 inflammasome in an ovalbumin‐induced asthma model [32].

### ASC regulation by ubiquitination and phosphorylation

#### Ubiquitination and deubiquitination control ASC oligomerization

**Fig. 3:**
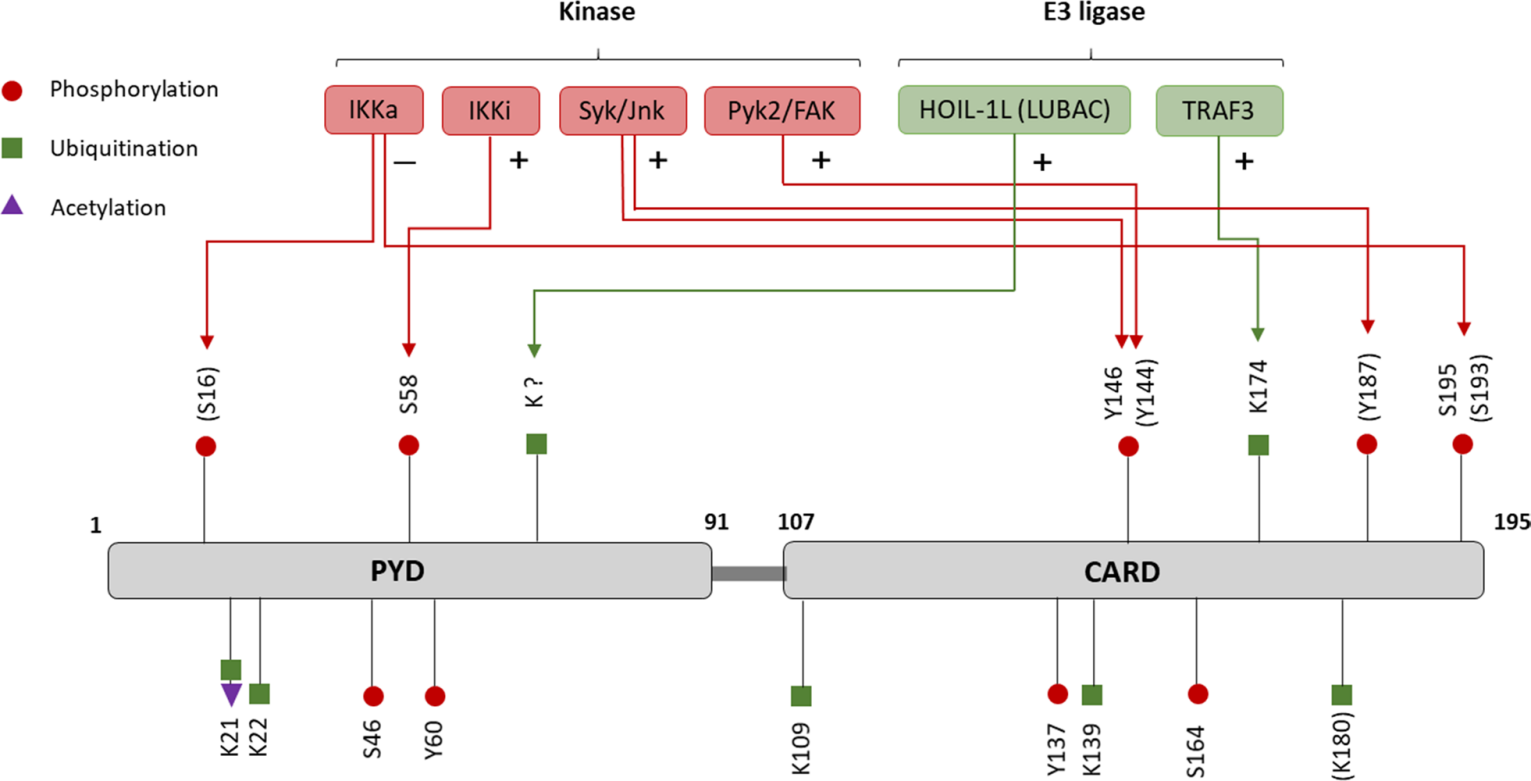
ASC/PYCARD PTMs and the enzymes responsible for their regulation. Empirically validated enzymes which are responsible for the PTM regulation of ASC/PYCARD. “+” indicates that the enzyme promotes the activation of ASC oligomerization, while “−” indicates repression of its function. Amino acid residues shown above refer to human ASC, those in brackets refer to mouse ASC residues

ASC/PYCARD has been shown to be subjected to K-63-linked poly-ubiquitination [33] and linear ubiquitination [34]. So far, among the 10 lysine sites in human or mouse ASC, 6 ubiquitination sites (K21, K22, K109, K139, K174 in human, K180 in mouse) have been identified by mass spectrometry or mutations[35] [36] (Fig. 3).

In resting cells, ASC is usually hypo-ubiquitinated and remains in an inactive state. Upon stimulation by LPS, ASC is ubiquitinated by the linear ubiquitin chain assembly complex (LUBAC), consisting of HOIL-1, HOIP, and SHARPIN. It has been reported that ASC is a direct substrate of HOIL-1, and its linear ubiquitination is required for the ASC oligomer formation [34] (Table 1, Fig. 3). Notably, deficiency of SHARPIN in mouse BMDMs impedes NLRP3 inflammasome activation through transcriptional regulation rather than ubiquitination [37]. In addition, upon activation by vesicular stomatitis virus (VSV), TNF receptor-associated factor 3 (TRAF3) induces K-63-linked poly-ubiquitination of ASC (K174) and positively regulates complex assembly. This process is controlled by mitochondrial antiviral-signalling protein (MAVS) on mitochondria, which is thought to provide a platform for the interaction between TRAF3 and ASC [33]. The importance of K174 on ASC in other activators-induced inflammasome activation needs to be elucidated.

In addition to ubiquitination, the deubiquitination process of ASC remains poorly understood, with only one DUB reported. Lee et al*.* showed that, ubiquitin specific peptidase 50 (USP50) directly binds to ASC and positively regulate ASC oligomerization by promoting deubiquitination of K-63-linked ubiquitin chains on the ASC protein [38] (Table 2). USP50 depletion markedly impedes the formation of ASC oligomer and IL-1β secretion in both human THP-1 macrophages and mouse BMDMs. Of note, USP50 is considered to be catalytically inactive because it lacks the conserved Asp/Asn residues that are required for catalytic activity [39]. Additional studies are therefore needed to explain the mechanistic details of USP50 on ASC regulation.

#### Phosphorylation and dephosphorylation regulate ASC localization and complex assembly

As an adaptor protein linking the NLRP3 sensor and the effector caspase-1, ASC plays a critical role in the assembly of the inflammasome complex. It has been reported that phosphorylation on ASC affects its oligomerization and localization during the priming and activation of NLRP3 inflammasome. So far, 9 phosphorylation sites have been identified on human or mouse ASC, namely (S16), S46, S58 [40], Y60 [41], Y137 [41], Y146 (Y144), S164, (Y187) and S195 (S193) (in human ASC, those in brackets refer to sites in mouse ASC) [36, 42]. The phosphorylation of S58 and Y144 were shown to positively regulate NLRP3 activation, while S16 and S193 phosphorylation exhibit inhibitory functions [43, 44] (Fig. 3, Table 3).

In resting cells, active IKKα consecutively phosphorylates ASC at S16 and S193 (in mouse ASC), which negatively regulates the activation via its interaction with ASC. Upon stimulation by LPS/ATP, IKKα dissociates from ASC. IKK-related kinase, IKKi, subsequently induces S58 phosphorylation on ASC and facilitates its redistribution from the nucleus to the cytoplasm, which in turn activates downstream signalling pathway [43] (Fig. 3, Table 3). Two groups have recently reported that phosphorylation of Y144 and Y187 (in mouse ASC) were indispensable for ASC oligomerization and subsequent caspase-1 recruitment [40, 44]. Syk and JNK were implicated to be involved in this phosphorylation process, with Syk suggested to be a new component of the NLRP3 complex [40]. Additionally, Syk-induced phosphorylation of Pyk2 was found to increase upon nigericin treatment, leading to the co-localization of phosphorylated Pyk2 with ASC speck. P-Pyk2 directly binds to ASC and promotes its phosphorylation at Y146 (in human ASC, Y144 in mouse ASC), which can be blocked by the Pyk2/FAK inhibitor (PF-431396) [45] (Table 5). In summary, these findings highlight the dynamic regulation of both priming and activation process by ASC phosphorylation.

**Table 5:** Inhibitors that target enzymes involved in PTM regulation of the NLRP3 inflammasome

Inhibitor	Target	Inhibition mechanism	Selectivity	Clinical status	Diseases	Ref.
SP600125	JNK1	JNK-1-induced NLRP3 S198 (S194) phosphorylation	JNK1, 2, 3	–		[29]
CRT0066101	PKD	PKD-induced NLRP3 S295 (S291) phosphorylation	PKD1, 2, 3	–		[31]
Gö 6976	PKD	PKD-induced NLRP3 S295 (S291) phosphorylation	PKD1, 2, 3	–		[31]
CID755673	PKD	PKD-induced NLRP3 S295 (S291) phosphorylation	PKD1, 2, 3	–		[31]
kb NB 142–70	PKD	PKD-induced NLRP3 S295 (S291) phosphorylation	PKD1, 2, 3	–		[31]
R406	Syk	Syk-induced ASC Y146 (Y144) phosphorylation	Syk	Phase I		[40, 57]
Piceatannol	Syk	Syk-induced ASC Y146 (Y144) phosphorylation	Syk	–		[67]
PF-562271	PYK2/FAK	PYK2-induced ASC Y146 (Y144) phosphorylation	PYK2/FAK	–		[45]
PF-431396	PYK2/FAK	PYK2-induced ASC Y146 (Y144) phosphorylation	PYK2/FAK	–		[45]
PF-573228	FAK	unknown	FAK	–		[45]
LFM-A13	BTK	ASC oligomerization	BTK			[61]
CGI1746	BTK	ASC oligomerization	BTK			[62]
Ibrutinib	BTK	ASC oligomerization	BTK	Approved	Mantle cell lymphoma, chronic lymphocytic leukemia	[61, 62]
G5	BRCC3	BRCC-induced NLRP3 deubiquitination	DUB pan inhibitor	–		[9]
HBX19818	USP7	USP7-induced NLRP3 deubiquitination	USP7	–		[8]
P005091	USP7	USP7-induced NLRP3 deubiquitination	USP7	–		[8]
P22077	USP7/USP47	USP7/USP47-induced NLRP3 deubiquitination	USP7/USP47	–		[8]

Amino acid residues shown above refer to human NLRP3, those in brackets refer to mouse NLRP3 residues

### PTM regulation of caspase-1

#### Ubiquitination and deubiquitination of caspase-1

**Fig. 4:**
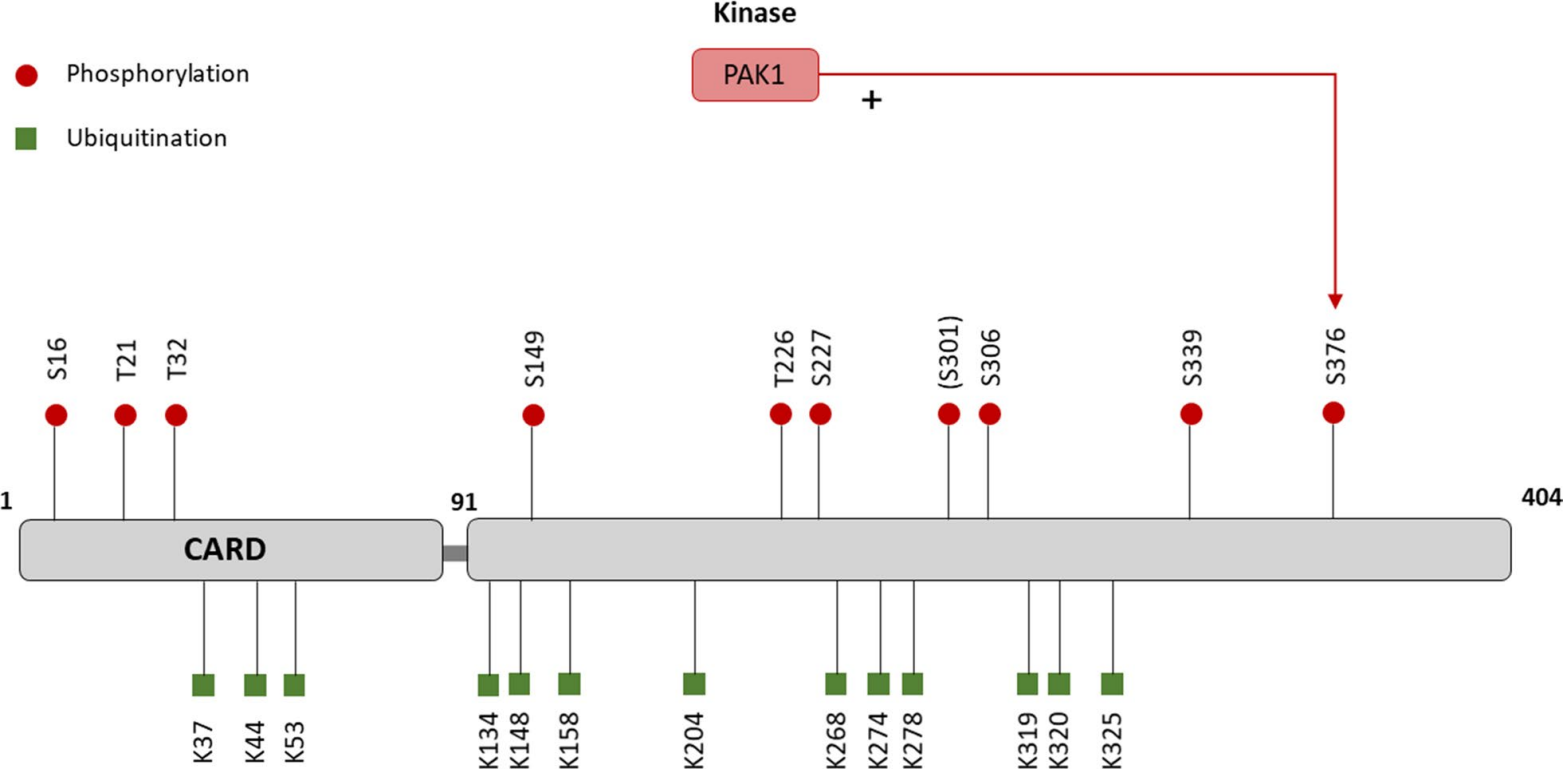
Caspase-1 PTMs and the enzymes responsible for their regulation. Empirically validated enzymes which are responsible for the PTM regulation of Caspase-1. “+” indicates that the enzyme promotes the activation of Caspase-1, while “−” indicates repression of its function. Amino acid residues shown above refer to human Caspase-1, those in brackets refer to mouse Caspase-1 residues

So far, 13 ubiquitination sites (K37, K44, K53, K134, K148, K158, K204, K268, K274, K278, K319, K320 and K325) have been identified in human caspase-1 [42], although the role of ubiquitination for caspase-1 activity is not yet fully understood (Table 1, Fig. 4). Two reports showed contradictory functions of inhibitor of apoptosis proteins (IAPs, including XIAP, cIAP1, cIAP2) on inflammasome activation. One study shows that cIAP1 /cIAP2 interact with caspase-1 and mediate its K-63-linked poly-ubiquitination, which positively regulates NLRP3 activation [46]. Conversely, Labbe et al*.* showed that defective IAPs activate caspase-1-dependent and independent IL-1β production, which both require RIP3 kinase [47].

#### Phosphorylation and dephosphorylation of caspase-1

Although there are 10 phosphorylation sites (S16, T21, T32, S149, T226, S227, S306, S339, and S376 in human caspase-1, S301 in mouse caspase-1) [42, 48] identified through mass spectrometry, only one (S376) was reported to function in the regulation of NLRP3 inflammasome (Table 3, Fig. 4). Basak et al*.* found that *Helicobacter pylori* LPS alone was able to trigger the maturation and release of IL-1β via Rac1/PAK1 signalling pathway. Upon stimulation, PAK1 interacts with and directly phosphorylates caspase-1 at S376, which in turn leads to its cleavage and subsequent IL-1β secretion [49]. In contrast to NLRP3 and ASC, the phosphorylation and dephosphorylation of caspase-1 remain poorly understood.

### Other modifications regulating inflammasome components

#### Ubiquitin-like conjugation (Sumoylation, Neddylation)

In resting cells, NLRP3 is Sumoylated by SUMO E3-ligase MAPL. Upon stimulation by nigericin, MAPL dissociates from NLRP3, and subsequently, Sumoylation level of NLRP3 is markedly reduced. Furthermore, knockdown of SUMO de-conjugating enzymes SENP6 and SENP7 selectively repress ASC oligomerization, caspase-1 cleavage and IL-1β secretion in a NLRP3-dependent manner, strongly indicating Sumoylation involvement in NLRP3 inflammasome activation [16]. In addition to Sumoylation, Neddylation is also reported to be required for efficient caspase-1 activation. Overexpression of NEDD8 dramatically enhanced IL-1β production in the reconstituted NLRP3 inflammasome model in HEK293 cells, while defective Neddylation, either via using inhibitor (MLN4924) or siRNA knockdown, resulted in inhibition of NLRP3 activation [17]. Even though NEDD8 is shown to bind to and co-localize with caspase-1, there is still no evidence that caspase-1 is the direct substrate. Additional studies are needed to understand the roles of these UBL conjugations in the regulation NLRP3 inflammasome activation.

#### Acetylation as a switch for NLRP3 activation

Recently, NLRP3 was shown to be acetylated at K21, K22 and K24 (in mouse, K23, K24 and K26 in human NLRP3) promoting the assembly and activation of NLRP3 inflammasome, controlled by the NAD-dependent protein deacetylase sirtuin-2 (SIRT2). LPS and ATP were shown to promote the acetylation level on NLRP3, and in turn increase the production of IL-1β, which was completely repressed by K21/22/24R mutations [11]. The SIRT2-NLRP3 axis has also been implicated in the aging-associated chronic inflammation and insulin resistance [13].

#### ADP-ribosylation mediated by pathogen-derived factors

Several pathogen-derived proteins have been reported to act as activators for NLRP3 inflammasome assembly. It was shown that a *Mycoplasma pneumonia-*derived toxin CARDS interacts with and activates NLRP3 inflammasome, dependent on its ADP-ribosyltransferase activity. Upon stimulation by CARDS, NLRP3 was found to be ADP-ribosylated, suggesting ADP-ribosylation of NLRP3 is a functional PTM for its positive regulation. Further studies are needed to understand the detailed molecular mechanism [14].

## Crosstalk between different PTMs

While single PTM regulation of NLRP3 inflammasome has been studied, the interplay between different PTMs is just beginning to be evaluated, largely expanding the complexity of NLRP3 regulation. Crosstalk between multiple PTMs can be either positive or negative, and occur at different levels [50, 51]. In the case of NLRP3 activation, both ubiquitination and phosphorylation are shown to be essential modifiers of inflammasome activity during the priming process. Song et al*.* found that NLRP3 S194 phosphorylation promotes its interaction with the deubiquitinase BRCC3, which in turn induces the deubiquitination of NLRP3 and its subsequent activation. Deubiquitination can be prevented by either S194A mutation or using a JNK agonist (anisomycin) [29], which suggests that NLRP3 phosphorylation might be an upstream regulator of NLRP3 deubiquitination. This is a good example of crosstalk by PTM regulation through creating binding sites for protein–protein interaction.

Another example for the crosstalk between phosphorylation and ubiquitination is the Src-family kinases (SFKs)-Cbl-Pyk2 axis in the regulation of ASC phosphorylation. Phosphorylated Pyk2 was shown to co-localize with ASC upon activation and promote ASC phosphorylation that is required for subsequent oligomerization [45]. The tyrosine kinase SFK was shown to mediate Cbl Y371 phosphorylation, which in turn negatively regulates Pyk2 via poly-ubiquitination and proteasome-dependent degradation [52]. Here, the interplay is achieved mainly through regulation of E3 ligase activity by phosphorylation.

Additionally, in the case of NLRP3, K689 can be modified by both ubiquitination and sumoylation. It was reported that, poly-ubiquitination of K689 represses the formation of NLRP3 inflammasome [7, 23], and sumoylation of the same lysine site is also important for the maintenance of the resting status [16]. The function of these two PTMs has been reported separately by three groups, but the relationship between them still remains unclear.

In summary, the combinatorial control of NLRP3 complex by multiple PTMs is emerging as an essential early event for the priming process, while more studies are needed to decipher the PTM codes and explain the crosstalk between them.

## PTMs involved in NLRP3 inflammasome translocation

PTMs are able to affect protein biological functions in multiple ways, including regulation of protein–protein interaction, functional activities, stability (half-lives), folding and subcellular localization. An important focus on NLRP3 inflammasome research is the translocation of inflammasome components between different subcellular compartments during the priming and activation process [53].

PTM regulation of either NRLP3 or ASC was reported to affect their localisation. For example, upon stimulation, the increase of diacylglycerol (DAG) in Golgi induces recruitment of PKD, which in turn phosphorylates NLRP3 (at S295) in MAMs. This leads to the release of phosphorylated NLRP3 from MAMs and subsequent assembly of the inflammasome complex in the cytosol [31].

Another example is the translocation of ASC induced by IKKi-mediated phosphorylation. Mechanistically, under resting condition, IKKα constitutively interacts with ASC in the nucleus and prevents the complex from hyper-activation. Upon stimulation with LPS and ATP, ASC is released from IKKα and is phosphorylated at S58 by IKKi, which facilitates its translocation from the nuclear to the perinuclear area resulting in complex assembly [43]. However, the cellular localisation of either NLRP3 or ASC is still a controversial topic. No consensus model exists for NLRP3 complex translocation perhaps differing in different cell models and with stimulation conditions.

## Pharmacological inhibitors targeting enzymes that write, read and erase inflammasome PTMs

As NLRP3 inflammasome is linked to multiple diseases, there is growing interest in exploring the therapeutic potential of direct or indirect targeting this complex. A small number of selective and potent NLRP3 inhibitors (such as MCC950 [54], tranilast [55], and CY-09 [56]) directly targeting the core sensor protein NLRP3 have been shown to be effective preclinically in inflammatory or auto-inflammatory diseases, recently reviewed elsewhere [1]. In the past decade, the enzymes (such as DUBs and kinases) involved in PTM control of NLRP3 complex are emerging as attractive drug targets. Here, we mainly focus on the small-molecule inhibitors that target these enzymes (Table 5).

Targeting the JNK pathway has long been considered as a strategy for treatment of inflammatory disease. SP600125, a selective and potent anthrapyrazolone inhibitor of JNK1, 2 and 3, was first identified and characterized as a JNK inhibitor in a high-throughput biochemical screening in 2001 [50]. SP600125 was shown to exhibit robust inhibitory effect in the expression of inflammatory genes such as COX-2, IL-2, IFN-γ, and TNFα through inhibition of c-Jun phosphorylation [50]. Recently, SP600125 was reported to inhibit nigericin-induced caspase-1 cleavage and IL-1β maturation in LPS-primed immortalized BMDMs [34]. Mechanistically, SP600125 directly inhibits JNK kinase activity, and in turn prevents JNK-induced NLRP3 S194 phosphorylation, which is an essential priming event [34].

As mentioned above, PKD kinases have been recently implicated in the regulation of NLRP3 inflammasome formation [36]. Four PKD inhibitors (CRT0066101, Gö 6976, CID755673, and kb NB 142–70) were reported to block NLRP3 inflammasome activation. Overexpression of PKD was shown to be sufficient for NLRP3 phosphorylation and activation, which highlights its importance in controlling the activation pathway.

The Syk-Pyk2 pathway has been shown to be involved in the phosphorylation regulation of NLRP3 inflammasome [40, 57]. R406, a potent and selective Syk inhibitor, was first discovered as a specific inhibitor for Fc epsilon RI (FcεRI) pathway [58], which is initiated by IgE binding and controls the production of multiple allergic mediators and cytokines. Gross et al. found that Syk deletion or pharmacological inhibition through R406 can completely block the production of IL-1β in response to fungal stimulation (*Candida albicans)* [57]. Another recent study also indicated that R406 was able to diminish ASC oligomerization through inhibition of ASC phosphorylation in ATP-or nigericin-induced NLRP3 inflammasome activation model [40]. In addition to R406, the Syk inhibitor piceatannol was also shown to inhibit IL-1β maturation and secretion stimulated by hemozoin in THP-1 macrophages [40]. Proline-rich tyrosine kinase 2 (Pyk2), which is downstream of Syk, was shown to directly phosphorylate ASC at Tyr146 and promote ASC oligomerization, effects that were prevented by PF-562271 and PF-431396 (Pyk2/FAK dual inhibitors). Of note, the FAK-specific inhibitor (PF-573228) was also able to interfere with the IL-1β production, although the mechanism of action requires further investigation [45].

As a key modulator downstream of B cell receptor signalling pathway, Bruton’s tyrosine kinase (BTK) is emerging as a promising drug target in B cell malignancies and autoimmune disorders [59]. The FDA-approved first-in-class BTK inhibitor ibrutinib (PCI-32765) is now used for mantle cell lymphoma, Waldenstrom's macroglobulinaemia and chronic lymphocytic leukemia [60]. Two groups have recently shown that BTK is critical modulator of NLRP3 inflammasome activation [61, 62]. Pharmacological inhibition of BTK by ibrutinib, LFM-A13 or CGI1746 precludes caspase-1 cleavage and IL-1β maturation, without changing the expression level of either pro-capase-1 or pro-IL-1β [18] [51]. Due to potential off-target effects, irreversible covalent BTK inhibitors, such as ibrutinib, are not approved by FDA for long-term treatment of autoimmune disorders [60]. Thus, the increasing number of reversible BTK inhibitors, such as CGI1746, holds promise for the IL-1β-driven inflammatory diseases [63].

G5 (also known as 3,5-bis((4-nitrophenyl)methylene)-1,1-dioxide,tetrahydro-4H-thiopyran-4-one), a pan DUB inhibitor, was initially identified in a screening for compounds that are able to trigger caspase and induce apoptosis [51]. It was later shown to specifically inhibit NLRP3 inflammasome activation but not AIM2 or NLRC4 inflammasome. This led to the discovery of BRCC3 as a critical deubiquitinase in the regulation of NLRP3 activation [14]. The BRCC3-dependent mechanism opens new avenues for targeting the NLRP3 inflammasome, and the development of BRCC3 selective inhibitors may prove to be potentially beneficial in a clinical context.

In addition to the DUB inhibitor G5, another study found that P22077, an inhibitor of USP7/USP47, precludes the formation of ASC oligomers, which subsequently inhibits caspase-1 and IL-1β maturation [8]. Two other USP7 inhibitors (P005091 and HBX19818) have also been shown to have similar inflammasome inhibitory effects [8]. Notably, although USP7/USP47 were shown to regulate inflammasome activation, it remains unclear whether NLRP3 is their direct substrate. Further studies are warranted to clarify the effect of USP7/USP47 inhibitors as inflammasome inhibitors.

## Conclusions and future perspectives

The uncontrolled activation of NLRP3 inflammasome has huge impact on multiple inflammatory and autoinflammatory diseases, making NLRP3 a promising therapeutic target. The increasing complexity of NLRP3 inflammasome regulation is partly ascribed to the post-translational regulation of its components. Although the discoveries of multiple PTMs on NLRP3/ASC/capase-1 and the enzymes responsible for these PTM regulation have advanced our knowledge of single PTM function in controlling NLRP3 inflammasome activation, a few important questions remain unanswered, including the interplay of multiple PTM regulation in sequential orders, and the identity of PTM enzyme readers, writers and erasers. In addition to NLRP3, ASC and caspase-1, other proteins, such as NIMA- related kinase 7 (NEK7) [64] and BTK [61, 62, 65], have been reported to be core components in the complex. Most recently, the phosphorylation of NEK at S204 by polo-like kinase 4 (PLK4) was found to attenuate the NLRP3-NEK7 interaction, thus inhibiting inflammasome activation. Interestingly, this phosphorylation process relies on the deubiquitylation of PLK4 by ubiquitin specific protease CYLD [68]. It provides another example of PTM crosstalk in inflammasome regulation. For BTK, there is no evidence that its kinase activity or post-translational modifications contribute to complex assembly. Further studies are needed to clarify the significance of PTM regulation for these newly identified NLRP3 inflammasome components.

The direct NLRP3 inhibitor MCC950 (also known as CRID3 or CP-456,773) has not progressed towards the clinic. Therefore, there is still a great need to identify therapeutic intervention points in the pathway to modulate the NLRP3 inflammasome [66]. With the refinement of our understanding of post-translational control during NLRP3 inflammasome activation, pharmacological inhibitors targeting enzymes that write, read and erase inflammasome PTMs will become promising candidates for those NLRP3-related inflammatory diseases.

## Data Availability

Not applicable.

## Abbreviations

AECs: Airway epithelial cells
ARIH2: Ariadne homolog 2
ASC: Apoptosis-associated speck-like protein containing a CARD
BMDMs: Bone marrow derived macrophages
BTK: Bruton’s tyrosine kinase
CBLB: Casitas B-lineage lymphoma proto-oncogene b
CINCA: Chronic infantile neurologic cutaneous and articular syndrome
CUL1: Cullin1
DAG: Diacylglycerol
DAMPs: Pathogen-associated molecular patterns
DUB: Deubiquitinase
EphA2: Ephrin type-A receptor 2
FBXL2: F-box/LRR-repeat protein 2
FcεRI: Fc epsilon RI
IAPs: Inhibitor of apoptosis proteins
IKKi: Inducible I kappa-B kinase
IKKα: Inhibitor of nuclear factor kappa-B kinase subunit alpha
JNK1: Jun N-terminal kinase-1
LPS: Lipopolysaccharides
LUBAC: Linear ubiquitin chain assembly complex
MAMs: Mitochondria-associated membranes
MAVS: Mitochondrial antiviral-signalling protein
NEK7: NIMA- related kinase 7
NLRP3: NACHT, LRR and PYD domains-containing protein 3
PAMPs: Damage-associated molecular patterns
PTMs: Post-translational modifications
PTPN22: Tyrosine-protein phosphatase non-receptor type 22
RNF125: RING finger protein 125
SCF: SKP1-cullin-F-box protein
SIRT2: NAD-dependent protein deacetylase sirtuin-2
TLR: Toll-like receptor
TRAF3: TNF receptor-associated factor 3
TRIM31: Tripartite motif-containing protein 31
USP47: Ubiquitin carboxyl-terminal hydrolase 47
USP50: Ubiquitin specific peptidase 50
USP7: Ubiquitin carboxyl-terminal hydrolase 7
VSV: Vesicular stomatitis virus
